# A nationwide survey of *Leishmania infantum* infection in cats and associated risk factors in Italy

**DOI:** 10.1371/journal.pntd.0007594

**Published:** 2019-07-15

**Authors:** Roberta Iatta, Tommaso Furlanello, Vito Colella, Viviana Domenica Tarallo, Maria Stefania Latrofa, Emanuele Brianti, Paolo Trerotoli, Nicola Decaro, Eleonora Lorusso, Bettina Schunack, Guadalupe Mirò, Filipe Dantas-Torres, Domenico Otranto

**Affiliations:** 1 Dipartimento di Medicina Veterinaria, Università degli Studi di Bari, Bari, Italy; 2 Clinica Veterinaria San Marco, Veggiano, Padova, Italy; 3 Faculty of Veterinary and Agricultural Sciences, University of Melbourne, Parkville, Australia; 4 Dipartimento di Scienze Veterinarie, Università degli Studi di Messina, Messina, Italy; 5 Dipartimento di Scienze Biomediche e Oncologia Umana, Università degli Studi di Bari, Bari, Italy; 6 Bayer Animal Health GmbH, Leverkusen, Germany; 7 Departamento de Sanidad Animal, Facultad de Veterinaria, Universidad Complutense de Madrid, Spain; 8 Department of Immunology, Instituto Aggeu Magalhães, Fundação Oswaldo Cruz (Fiocruz), Recife, Brazil; University of Queensland, AUSTRALIA

## Abstract

Though scantly investigated, *Leishmania infantum* infection and clinical cases of leishmaniasis in cats have been recently reported in several countries of the Mediterranean basin, with large variability in prevalence data. A major limitation in the comparability of the data available is attributed to the differences in diagnostic techniques employed and cat populations sampled. The aim of this study was to assess the prevalence of *L*. *infantum* infection in owned cats across Italy by serological and molecular tests and the identification of potential risk factors. Blood samples from 2,659 cats from northern (n = 1,543), central (n = 471) and southern (n = 645) Italy were tested for antibodies against *L*. *infantum*, by an immunofluorescence antibody test and for the parasites’ DNA, by real-time PCR. Samples were additionally screened for feline leukemia virus (FeLV) and feline immunodeficiency virus (FIV) proviral DNAs. An overall cumulative *L*. *infantum* prevalence of 3.9% was recorded by serology (3.3%) and/or qPCR (0.8%), with a higher rate (10.5%) in southern Italy. The risk of *L*. *infantum* infection in cats was significantly associated to the geographical areas (South *vs* North and Centre; p<0.0001), age class (from 19 months to 6 years old *vs* ≤18 months old, p = 0.0003), neutering status (not neutered *vs* neutered, p = 0.0028) and FIV infection (p = 0.0051).Though the role of cats in the epidemiology of *L*. *infantum* is still debated, our findings indicate that cats are exposed to and/or infected by this protozoan, mainly in endemic regions of Italy. Hence, a standardization of procedures for a prompt diagnosis of *L*. *infantum* infection in cats and for screening cat population is crucial for a better understanding of the epidemiology of feline leishmaniasis, and of the potential role of cats in the transmission cycle of zoonotic visceral leishmaniasis.

## Author summary

Zoonotic visceral leishmaniasis is a potentially fatal parasitic disease, which is caused by *Leishmania infantum*. Its distribution is associated with the occurrence of the sand fly vectors and reservoir hosts. Since *L*. *infantum* infection can occur in cats with clinical or subclinical outcomes, the role of cats in the epidemiology of zoonotic visceral leishmaniasis needs to be thoroughly assessed. This study aimed to evaluate the prevalence and associated risk factors for infection with *L*. *infantum* in a large subset of cats across Italy, a known endemic area with records of human cases of visceral leishmaniasis. Serum and blood samples from 2,659 cats from northern (n = 1,543), central (n = 471) and southern (n = 645) Italy were tested for antibodies against *L*. *infantum* and parasites’ DNA, respectively. A cumulative *L*. *infantum* prevalence of 3.9% was recorded by serology (3.3%) and/or real-time PCR (0.8%). The risk of *L*. *infantum* infection in cats was associated to the geographical areas, age class, neutering status and feline immunodeficiency virus infection. These findings reveal that cats are exposed to and/or infected by this protozoan across the country, warranting further investigation to assess their role in the epidemiology of zoonotic visceral leishmaniasis to refine surveillance and prevention strategies against this veterinary and medically important ailment.

## Introduction

Amongst vector-borne zoonoses, visceral leishmaniasis (VL) by *Leishmania infantum* is a major global disease potentially fatal to humans. VL is one of the most important threats among the neglected tropical diseases causing an estimated 300,000 new cases and about 20,000 deaths in humans each year [1]. Its distribution is associated with the occurrence of the phlebotomine sand fly vectors of the genus *Phlebotomus* spp. and *Lutzomyia* spp., in the Old and New World, respectively. Developing countries take the brunt of VL considering that malnutrition and low hygienic conditions represent risk factors for the spreading of the infection in human patients [2]. Dogs are the main reservoir hosts of the parasite [3] with usually more than 30% seropositive animals in endemic areas [4]. Other animal species, such as cats and some wild animals (e.g., foxes and hares) have been implicated in the epidemiology of the infection [5,6], with hares involved as reservoir hosts in the outbreak of VL in south-western Madrid, Spain [7]. Where canine leishmaniasis (CanL) is endemic, cats are often exposed to *L*. *infantum*, with seroprevalence ranging from 0.7% to 30% according to animal life style and diagnostic technique used [8]. In a study conducted in southern Italy (i.e., the Aeolian Islands, Sicily), a cumulative *L*. *infantum* serological and molecular prevalence of 25.8% in cats was reported [9]. Differences in the feline immune function, such as an effective Th1 immunity which often allows spontaneous resolution of lesions, may play a role for the reduced clinical signs in infected cats [10] resulting in subclinical forms with only few reports of overt illness, mainly characterised by skin lesions and lymphadenomegaly [8]. Though cats are exposed to sand fly bites [11], the overall prevalence of *L*. *infantum* infection is generally lower than in dogs living in the same areas [8,9,12,13]. Consequently, feline leishmaniasis (FeL) has been for long time disregarded by veterinary practitioners and parasitologists and, as a result, the current distribution of this disease may be underestimated. Examples of neglected zoonotic VL are well embodied by the recent first report of *L*. *infantum* in dogs and a cat from Bosnia and Herzegovina, where human leishmaniasis is known to occur in people visiting the country and in the local population [14]. Further, the subclinical presentation of FeL makes the diagnosis of the infection a complex task [8]. Concomitant infections with viral agents such as feline leukemia virus (FeLV), feline immunodeficiency virus (FIV), feline coronavirus (FCoV) and the protozoon *Toxoplasma gondii* have been diagnosed with FeL [13,15,16]. Since the first description of FeL in a domestic cat (*Felis silvestris catus*) [17], the number of reports of clinical cases and prevalence of *L*. *infantum* infection in cats have steadily increased in endemic areas, such as in the Mediterranean basin [12,13,18–20], the Middle East [21] and Brazil [22]. However, these data have been gained using different diagnostic methodologies since there is currently no consensus about the method of choice for diagnosing FeL. Serological [i.e., immunofluorescence antibody test (IFAT) and enzyme-linked immunosorbent assay (ELISA)], and molecular (e.g., real time-PCR, qPCR) tests are primarily employed for the diagnosis of FeL either for clinical and research purposes [9,23] though few investigations have used multiple tests in combination [9,15,24]. CanL is endemic in Italy with up to 40% of *L*. *infantum* infected dogs in the highly endemic regions of south-central Italy [25] where the infection in cats has also been recorded [9,23,26]. However, the lack of information about the distribution of *L*. *infantum* subclinical infections in cats is an hindrance to a clear understanding of the role of cats in the epidemiology of zoonotic VL in endemic areas. In this study we assessed the prevalence of *L*. *infantum* infection in a large number of cats across Italy by serological and molecular tests and identified potential risk factors for FeL.

## Materials and methods

### Ethics statement

From June 2017 to August 2018, serum and blood samples of cats were sent from six veterinary analysis laboratories distributed throughout Italy, to the Parasitology Unit of the Department of Veterinary Medicine, University of Bari (Italy) for serological and molecular testing. Samples were originally received for animal’s health check analyses. The protocol of this study was approved by the ethical committee of the Department of Veterinary Medicine of the University of Bari, Italy (Prot. Uniba 7/17).

### Sample collection

Blood and serum samples were obtained from 2,659 cats living in the North (n = 1,543), the Centre (n = 471) and the South (n = 645) of Italy. Animal data (i.e., age, sex, breed, neutering status and the owner’s province) were recorded. Cats were grouped according to age in younger than 18 months old (group 1, G1), between 19 months and 6 years old (group 2, G2) and elder than 6 years (group 3, G3).

### Serological testing

Serum samples were tested for anti-*L*. *infantum* antibodies with a slightly modified IFAT protocol previously described in Otranto *et al*., 2009 [27]. In particular, after the incubation of serum samples and fluoresceinated rabbit anti-cat immunoglobulin G (IgG) the slides were washed by immersion in phosphate-buffered saline three times for 10 min each by shaking. In addition, the conjugated anti-cat IgG was diluted 1:50 (Sigma-Aldrich, Germany). Serum samples from a cat positive for *L*. *infantum* by cytological and molecular analyses, and from 10 healthy cats living in a non-endemic area (Westbrook, Maine, USA), were used as positive and negative controls, respectively. Samples were scored as positive when they produced a clear cytoplasmic and membrane fluorescence of promastigotes from a cut-off dilution of 1:80, as recommended by current LeishVet guidelines [8]. Positive sera were titrated by serial dilutions until negative results were obtained.

### Molecular testing

Genomic DNA was extracted from blood using the GenUP DNA Kit (Biotechrabbit, Germany), following the producer’s recommendations. The detection of a fragment (120 bp) of *L*. *infantum* kDNA minicircle was achieved by qPCR, using primers, probes and protocol described elsewhere [28]. For all qPCR tests, DNA extracted from blood samples of a cat positive to *L*. *infantum* by cytological examination (i.e., positive control) and of 10 healthy cats living in a non-leishmania-endemic area (i.e., Westbrook, ME 04092 USA) (i.e., negative control) were included. Samples were scored as positive when a threshold cycle less than 37 was recorded. FeLV and FIV proviral DNAs were tested using primers and protocol previously described [29,30].

### Mapping and statistical analysis

The minimum sample size was calculated based on the following assumptions: infinite population size; confidence level of 95%; expected prevalence values of 20%, 10% and 5% according to provenance from South, Central and North Italy, respectively [8,9]; maximum accepted error 5% (South Italy), 3% (Central Italy) and 2% (North Italy). The location of *L*. *infantum* positive cats was geo-referenced using a geographical information system (GIS, ArcGIS version 10.3 ESRI), according to the owner’s province. Categorical data were summarized as count and percentage. Comparisons between independent groups were performed by chi-square test. Concordance between IFAT and qPCR was evaluated by McNemar test for paired data and Cohen’s K was determined as a measure of concordance. The odds ratio and its 95% confidence interval of *L*. *infantum* infection were calculated for each level of the categorical variables through a univariate and multivariate logistic regression analyses to evaluate the association between the risk of *L*. *infantum* infection by IFAT or qPCR and independent variables (i.e., sex, age class, neutering status and geographic area). In the multivariate analysis FIV and FELV results were included for evaluating the association between *L*. *infantum* and FIV or/and FELV infections. The age is reported in months or years and is summarised as mean and standard deviation and comparisons between independent groups were evaluated with t-student test. Analyses were performed with the software SAS V9.4 for a personal computer. A p-value<0.05 was considered for statistical significance.

## Results

The number of sampled cats (i.e., 2,659) enrolled from northern (n = 1,543), central (n = 471) and southern (n = 645) Italy exceeded the minimum sample size (i.e., 457, 385 and 246 samples from northern, central and southern Italy, respectively). Owned cats enrolled in the study (i.e., n = 1,302 males and n = 1,357 females) aged from 1 month to 21.3 years (mean 8.3 years, median 9 years). Of these, 359 (13.5%) were less than 18 months old (G1), 599 (22.5%) between 18 months and 6 years old (G2) and 1,701 (64%) elder than 6 years (G3). Most of the animals were common European breed (n = 2,329, 87.6%) and neutered (n = 2,275, 85.5%) (Table 1). The prevalence of *L*. *infantum* infection by serology and/or molecular test in association with age, sex, breed, neutering status and cat origin is reported in Table 1. Overall, 104 (3.9%) cats were positive to *L*. *infantum* by IFAT (88/2,659, 3.3%) and/or by qPCR (22/2,659, 0.8%) with statistically significant (McNemar test p<0.0001) difference and poor concordance between the techniques, K = 0.097 (95% CI: 0.0176–0.1766). Out of these 104 positive cats, 6 (5.8%) were positive to both qPCR and IFAT with IgG titres of 1:80 (n = 3), 1:640 (n = 2) and 1:5120 (n = 1). Out of the 22 PCR positive cats, 16 (72.7%) were seronegative. Of the seropositive cats, 84.1% (74/88) had an antibody titre of 1:80, whereas in the remaining the titres varied from 1:160 to 1:5120 (Table 1). The prevalence of positive cats detected by serological and/or molecular tests in the different geographic areas was 10.5% (68/645) in the South, 2.3% (11/471) in the Centre and 1.6% (25/1543) in the North of Italy.

**Table 1:** Association between variables: Age, sex, breed, reproductive status and cat origin and the serological and molecular positivity for *Leishmania infantum*.

Variables	N	IFAT N (titre)	IFAT Pos (%)	qPCR Pos (%)	Total Pos (%)
**Age**					
≤18 months	359	5 (1:80); 2 (1:640)	7 (1.9)	2 (0.6)	8 (2.2)
18 months < 6 years	599	23 (1:80); 5 (1:160); 1 (1:640)	29 (4.8)	8 (1.3)	34 (5.7)
≥ 6 years	1701	46 (1:80); 5 (1:160); 1 (1:5120)	52 (3.0)	12 (0.7)	62 (3.7)
**Sex**					
Male	1302	43 (1:80); 5 (1:160)	48 (3.7)	11 (0.8)	58 (4.4)
Female	1357	31 (1:80); 3 (1:160); 5 (1:640); 1 (1:5120)	40 (2.9)	11 (0.8)	46 (3.4)
**Neutering status**					
neutered	2275	50 (1:80); 6 (1:160); 2 (1:640); 1 (1:5120)	59 (2.3)	22 (1.0)	75 (3.3)
not neutered	384	24 (1:80); 4 (1:160); 1 (1:640)	29 (7.5)	0	29 (7.5)
Breed*					
Common European	2329	68 (1:80); 10 (1:160); 3 (1:640); 1 (1:5120)	82 (3.5)	20 (0.9)	96 (4.1)
Persian	93	2 (1:80)	2 (2.1)	2 (2.1)	4 (1.0)
Maine Coon	53	0	0	0	0
Siamese	36	2 (1:80)	2 (2.1)	0	2 (2.1)
Norwegian of the forest	20	1 (1:80)	1(5.0)	0	1 (5.0)
Chartreux	19	0	0	0	0
Siberian	19	1 (1:80)	1 (5.3)	0	1 (5.3)
Exotic Shorthair	16	0	0	0	0
British Shorthair	15	0	0	0	0
**Geographical origin**					
North	1543	20 (1:80)	20 (1.3)	5 (0.3)	25 (1.6)
Centre	471	5 (1:80); 1 (1:5120)	6 (1.3)	6 (1.3)	11 (2.3)
South	645	49 (1:80); 10 (1:160); 3 (1:640)	62 (9.6)	11 (1.7)	68 (10.5)
**Total animals**	2659	74 (1:80); 10 (1:160); 3 (1:640); 1 (1:5120)	88 (3.3)	22 (0.8)	104 (3.9)

*only breeds with ≥ 15 animals listed

The results of the univariate and multivariate logistic regression analyses are shown in Table 2. The risk of *L*. *infantum* infection in cats, in the multivariate model (i.e., accounting for sex and age class) was significantly associated with the geographical areas (p<0.0001). In particular, cats living in the South of Italy were related to higher risk of *L*. *infantum* infection than those in the North by the univariate (OR = 7.14; 95% CI: 4.48–11.49) and multivariate analysis (OR = 2.66, 95% CI: 1.59–4.44), and those in the Centre by the univariate analysis (OR = 4.93; 95% CI: 2.58–9.43). Animals positive to *L*. *infantum* (n = 104; 3.9%) aged from 6 months to 20.1 years old (mean 7.5 years, standard deviation 5 years), while negative cats aged from 1 month to 21.3 years (mean 8.4 years, standard deviation 5.4 years) old with no statistically significant difference between the two groups (t = 1.795, p = 0.0728). For cats in G2 (i.e., 19 months to 6 years old) a higher *L*. *infantum* prevalence (5.7%, 34/599) (χ^2^ = 6.806; *p* = 0.0333) was recorded. The multivariate regression analysis showed that these G2 cats were related to a higher risk of *L*. *infantum* infection than those in G1 (i.e., ≤18 months) (OR = 3.69; 95% CI: 1.65–8.27). The neutering status was a statistically significant factor associated with positivity to *L*. *infantum*. In particular, the risk of not neutered compared to neutered cats was OR = 1.76 (95% CI: 1.06–2.93) and statistically significant (p = 0.028). No statistical association was found between *Leishmania* infected cats and sex in neither the univariate (p = 0.016), nor the multivariate model (p = 0.3015). Equally no association was observed for the breed variable (univariate analysis: p = 0.1413; multivariate analysis: p = 0.9947). Overall, 115 and 101 cats were positive to FeLV (4.3%) or FIV (3.8%) respectively, of which 6.1% (7/115) for FeLV and 12.9% (13/101) for FIV were also positive to *Leishmania*. Out of the 13 cats coinfected with FeLV and FIV, three (23.1%) were also *Leishmania* positive. *Leishmania infantum* infection was significantly associated only to FIV infection resulting in the multivariable model with an OR = 2.65 (95% CI: 1.34–5.22, p = 0.0051).

**Table 2:** Odds ratio and 95% confidence interval from logistic regression analysis for cats positive to *Leishmania infantum* by IFATand/or qPCR.

		Univariate analysis				Multivariate analysis			
		OR	95% CI		p-value	OR	95% CI		p-value
Sex	F *vs* M	0.75	0.51	1.12	0.16	0.81	0.53	1.22	0.3015
Neutering status	Not-neutered *vs* neutered	2.39	1.54	3.73	0.0001	1.76	1.06	2.93	0.028
Geographical area	South *vs* North	7.14	4.48	11.49	<0.0001	2.66	1.59	4.44	<0.0001
	South *vs* Centre	4.93	2.58	9.43		0.72	0.51	1.03	
	North *vs* Centre	0.69	0.34	1.41		0.27	0.12	0.61	
Breed	Common European vs other	1.73	0.83	3.59	0.1413	0.99	0.46	2.14	0.9947
Age-class	G2 *vs* G1	2.64	1.21	5.77	0.0211	3.69	1.65	8.27	0.0003
	G3 *vs* G1	1.63	1.004	2.67		2.66	1.59	4.44	
	G2 vs G3	1.61	1.15	2.26		1.39	0.98	1.97	
FIV	Pos *vs* Neg	4.01	2.16	7.44	<0.0001	2.65	1.34	5.22	0.0051
FeLV	Pos *vs* Neg	1.64	0.74	3.61	0.22	1.25	0.53	2.93	0.6079

CI: 95% confidence interval; OR: odds ratio

**Fig 1:**
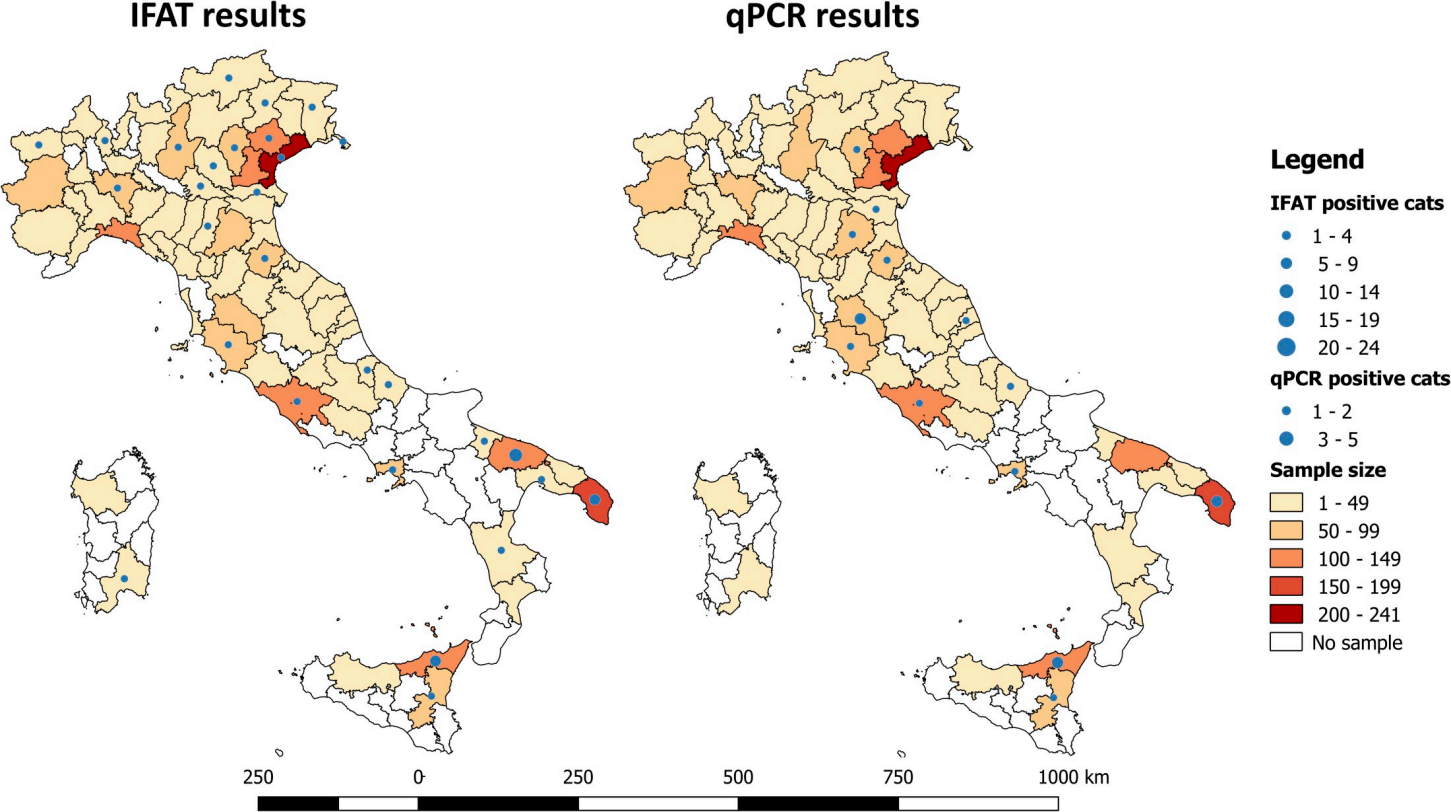
Geographical localization of areas, indicated by provinces, from where cats positive to *Leishmania infantum* by serological and molecular tests were collected. The location of *L*. *infantum* positive cats was geo-referenced using a geographical information system (GIS, ArcGIS version 10.3 ESRI).

At the GIS analysis, the highest number of cats (i.e., >100 cats) was sampled in 8 out of 83 provinces examined in the study area (i.e., n = 4 in the North, n = 1 in the Centre and n = 3 in the South of Italy). The geographical analysis identified the presence of three areas with a significant higher proportion of *L*. *infantum* seropositive cats, located in three provinces of southern Italy (i.e., 13.5% in Bari, 11.4% in Messina and 6.4% in Lecce; Chi-square test for linear trend = 23.4224, *df*  = 1, *p* = <0.0001) (Fig 1). In two of these three provinces (i.e., Messina and Lecce) a higher proportion of qPCR positive samples (3.8% and 1.6%, respectively) was also recorded (Fig 1).

## Discussion

The present study represents the largest epidemiological survey combining serological and molecular testing to assess the occurrence of *L*. *infantum* in cats to date.

The prevalence of FeL recorded by serology (3.3%) and qPCR (0.8%) indicates that cats are exposed to, or infected by, *L*. *infantum* throughout Italy. The overall seroprevalence of 3.3% is similar to that recorded in cats from central Spain (1.3–3.2%) and Portugal (2.8%) [12,31]. Higher seroprevalence data have been reported in cats from highly endemic regions of the Mediterranean basin for CanL, such as in southern Spain (28.3%) [19], central (16.3%) [26] and southern Italy (25.8%) [9] and Turkey (15.2%) [24]. It means that in areas where CanL is endemic, cats are likely to be more exposed to *L*. *infantum* as compared to areas where the prevalence of CanL is low [9]. Nonetheless, in our study different proportions of the population of infected animals were recorded in northern (1.6%) and centre (2.3%) versus southern (10.5%) Italy. Although *L*. *infantum* has spread throughout Italy [32], the southern and insular regions are still considered more endemic areas for human VL and CanL [25, 33], as a result of the favourable geographical climate conditions that allow the presence and abundance of sand fly vectors in the whole country (e.g., *Phlebotomus perniciosus*, *Phlebotomus perfiliewi*, *Phlebotomus ariasi*, and *Phlebotomus neglectus*) [25]. Accordingly, the occurrence of *L*. *infantum* in wild animals has been reported mainly in the centre and south of Italy [34–36].

In addition, current data available in the literature on the seroprevalence of FeL have been gained using different diagnostic methodologies and cut-off values, representing a limiting factor for data comparison. For example, for the IFAT, the percentage of seropositive animals was higher at a cut-off of 1:40 (i.e., 25.8% and 28.3%) [9,19], than that at 1:64 (i.e., 0.7%) [37], 1:100 (3.2%) [31] and 1:80 (3.3%; present study). Though, an IFAT cut-off (1:80) has been recommended by LeishVet group [8], more comprehensive and standardized protocols and ideally a defined gold standard procedure would allow a more consistent diagnosis of *L*. *infantum* infection in cats and contribute to a better understanding of the role of cats in the epidemiology of zoonotic VL.

The higher seroprevalence recorded in cats from southern (9.6%) versus central (1.3%) and northern (1.3%) regions of Italy suggests that cats are more exposed to *L*. *infantum* in the southern regions, as reported by previous serologic investigations conducted in Liguria and Tuscany (i.e., 0.9%) [18] and in Sicily and Calabria (southern Italy) (i.e., 6.9%) [38]. This is further supported by the GIS analysis that indicates a significant higher proportion of *L*. *infantum* positive cats in three areas of southern Italy. Likewise, though the distribution pattern of *L*. *infantum* infection is changing throughout the country as a result of many biological and ecological factors [32], CanL mainly occurs in southern Italy [25].

Since no information on the cats’ life style (i.e., outdoor/indoor access) and on sand fly population during the sample collection are available, the role of age and neutering status as risk factors for the occurrence of infections in cats, may be explained by differences in behaviour. Indeed, not neutered and older than 18 months cats are more prone to an outdoor life style than younger and neutered cats, due to more pronounced predatory instinct [39]. Therefore, these cats are more exposed to sand fly bites, which over time, results in a higher risk of *L*. *infantum* infection. Undoubtedly, the prevalence of cats positive for FeL at the molecular tests also depends on the cat life style and therefore exposure to sand fly bites with the higher number of animals positive to DNA recorded in feral and stray cats from Spain (i.e., 8.7% and 26%) [5,15] and Portugal (30.4%) [40].

*Leishmania infantum* DNA has been detected in blood samples of only a few cats (0.8%) as already reported in Portugal (0.3%) [41], northern (1.1%) [42] and southern Italy (2.1%) [9] and Cyprus (2.3%) [20]. This may suggest that blood is not the ideal tissue for molecular diagnosis of *L*. *infantum* infection in cats, as for dogs. Indeed, when comparing different matrixes for the molecular diagnosis of FeL, conjunctival swab (16.7%) and lymph node aspiration (11.7%) showed to be more sensitive than blood (7.8%) [37], as also reported for the diagnosis of CanL [27,43]. Though the number of samples positive with molecular diagnosis (n = 22) was limited in this study, the molecular positivity for *L*. *infantum* parallels the occurrence of higher seroprevalence in the examined animals, as reported previously [5,15]. The high percentage of molecular positivity and thus occurrence of *L*. *infantum* DNA in seronegative animals (72.7%, 16/22) might either indicate that these cats were at an early stage of the infection or endorse that they are less susceptible compared to dogs [10]. While there is a correlation between molecular and serological positivity in dogs infected for CanL [9,13], there is none between molecular and serological tests for FeL, supporting the different immune responsiveness of dogs and cats to *L*. *infantum*.

Longitudinal studies in cats could contribute in determining the course of *L*. *infantum* infection after natural exposure over time. Further, transmission studies with competent sand fly vectors could provide more information about the role of cats as reservoir hosts of *L*. *infantum*. So far, *P*. *perniciosus* has shown to feed on cats [44] and allow the developing of the parasite after the blood ingestion [11] thus potentially enabling further transmission of *L*. *infantum*. Since *P*. *perniciosus* is the main vector of *L*. *infantum* in Italy [45] and the most abundant phlebotomine sand fly species in some areas of southern Italy [46], further observations are needed to elucidate the role of naturally infected cats in sustaining the *L*. *infantum* life cycle.

The significant association between *L*. *infantum* and FIV infections (p = 0.0051) was previously reported [16,47] and indicates that immunosuppressive agents, such as FIV, impair the cellular immune response, thus increasing the risk for FeL. This correlation is supported by literature showing a high prevalence of FIV infection (i.e., 30%) in *L*. *infantum* infected cats [8]. Therefore, FIV and *L*. *infantum* coinfections might predispose animals to visceral forms, as an effect of the viral immunosuppression, as recognized in HIV seropositive patients [48].

In conclusion, differences in immune responses between dogs and cats and scant data on the ability of vectors in the transmission of *L*. *infantum* on natural infected cats, complicate the appreciation of the role of cats in *L*. *infantum* epidemiology. As future perspectives, the standardization of procedures for a prompt diagnosis of *L*. *infantum* infection and for screening cat populations is a crucial task for a better understanding of the epidemiology of FeL, and the role of cats as reservoir hosts. In addition, prevention measures for providing protection against the infection and treatment strategies for cats infected by *L*. *infantum* need to be further addressed.
